# Commercial Medical Colleges

**Published:** 1897-06

**Authors:** 


### Commercial Medical Colleges.

The sooner the medical profession of the United States determines to discourage all medical colleges in our midst, which are not endowed, or State institutions, and entirely independent of the fees of their students, the better. In other words, the profession, in its entirity, should determine that the "commercial medical college" must go.

Furthermore, a check should be put upon the dispensary abuse. The time is coming when there should be a combination upon the part of the members of the profession in favor of their own interests as against "wildcat" medical colleges, free dispensaries and other abuses, which have been developing in the profession during the last half century, and which will eventually pauperize the majority of doctors.

The truth of the matter is, if medical colleges were honest, they would announce in their advertisements that only one in ten of the applicants for admission into the profession is likely to succeed, unless they have inherited wealth or are in position to marry it.

It is a brutal and crying shame that innocent young men the country over are being inveigled, through the inducements offered by these medical colleges, into a profession which, year by year, is offering greater opportunities for starvation, and developing a spirit of commercialism which is to be deprecated.—*Med. Mirror.*

